# A Fast and Reliable Process to Fabricate Regenerated Silk Fibroin Solution from Degummed Silk in 4 Hours

**DOI:** 10.3390/ijms221910565

**Published:** 2021-09-29

**Authors:** Michael Wöltje, Arthur Kölbel, Dilbar Aibibu, Chokri Cherif

**Affiliations:** Institute of Textile Machinery and High-Performance Material Technology, Faculty of Mechanical Science and Engineering, Technische Universität Dresden, 01069 Dresden, Germany; arthur.koelbel@tu-dresden.de (A.K.); dilbar.aibibu@tu-dresden.de (D.A.); chokri.cherif@tu-dresden.de (C.C.)

**Keywords:** silk fibroin, degumming, ZnCl_2_, gel filtration

## Abstract

Silk fibroin has a high potential for use in several approaches for technological and biomedical applications. However, industrial production has been difficult to date due to the lengthy manufacturing process. Thus, this work investigates a novel procedure for the isolation of non-degraded regenerated silk fibroin that significantly reduces the processing time from 52 h for the standard methods to only 4 h. The replacement of the standard degumming protocol by repeated short-term microwave treatments enabled the generation of non-degraded degummed silk fibroin. Subsequently, a ZnCl_2_ solution was used to completely solubilize the degummed fibroin at only 45 °C with an incubation time of only 1 h. Desalting was performed by gel filtration. Based on these modifications, it was possible to generate a cytocompatible aqueous silk fibroin solution from degummed silk within only 4 h, thus shortening the total process time by 48 h without degrading the quality of the isolated silk fibroin solution.

## 1. Introduction

Silk fibroin is the structural protein of the filament of the silkworm *Bombyx mori* and combines properties such as high mechanical strength, biocompatibility, and biodegradability in vivo. The possibility of using silk fibroin in a wide variety of formats such as films, scaffolds, hydrogels, and micro- or nanoparticles for drug delivery, has led to many interesting applications in medical research (reviewed in detail in [1]). In addition, these properties also make silk fibroin attractive for the development of new advanced silk-based materials for soft bioelectronics. These soft systems include bioresorbable electronics for wearable sensors, electronic skins, and flexible energy devices (extensively reviewed in [2]).

To prepare a pure silk fibroin solution, the two protein components that make up the silk thread (fibroin as the structural component and adhesive sericin that helps to form a cocoon) must first be separated from each other. This process is called degumming. Laboratory-scale degumming includes many different methods such as the use of highly concentrated urea [3], organic acids such as boric acid sodium borate buffer, succinic acid [4], or citric acid [5], and various enzymes for enzymatic degumming [6].

The most common method for degumming is boiling raw silk fibers/cocoons in 0.02 M sodium carbonate (Na_2_CO_3_) buffer for 30 to 60 min followed by washing with water [7]. However, a prolonged boiling time or even a high Na_2_CO_3_ concentration leads to a change in molecular weight due to the fragmentation of silk fibroin and consequently to a reduction of the physical properties of the degummed silk fibers [3,8,9,10,11].

The second step in preparing a silk fibroin solution is to dissolve the degummed silk fibers. This dissolution step is inhibited by the strength of the hydrogen bonds and the hydrophobic nature of the β-sheet crystallites of silk fibroin. Therefore, aqueous or organic salt containing systems with high ionic strength are most suitable for complete dissolution of silk fibroin such as calcium chloride/formic acid (CaCl_2_/FA) [12], lithium salt solutions such as lithium thiocyanate (LiSCN) [3], and lithium bromide (LiBr-H_2_O) [7], calcium nitrate/methanol (Ca(NO_3_)_2_)/CH_3_OH) mixtures [13], N-methylmorpholine-N-oxide (NMMO) [14], ionic liquids [15,16,17] and the so-called Ajisawa’s reagent consisting of calcium chloride/water/ethanol (CaCl_2_/H_2_O/C_2_H_5_OH) [18]. The most used solvent systems for degummed silk fibroin fibers in the literature are 9.3 M LiBr-H_2_O and Ajisawa’s ternary solvent system. In the former, degummed silk fibers are dissolved for 4 h at 60 °C [19], whereas in the latter, 3 h at 65 °C is sufficient for complete dissolution [18]. For the subsequent regeneration of silk fibroin, the silk fibroin salt solution must first be desalted. The most common method to remove salt ions is to dialyze the highly viscous silk fibroin salt solution against ultrapure water for 2 days [19,20]. This very time-consuming procedure, which additionally consumes a lot of water, is also the main reason why there is no large-scale or industrial processing and application of the silk fibroin solution so far.

In summary, there is a need for research in the field of low degradation degumming, dissolution, and reproducible desalting methodology that would allow the isolation of native-like silk fibroin and could eventually be used for the future production of regenerated silk fibroin on an industrial scale.

Therefore, the aim of the present study was to first develop a gentler degumming method that allows starting the dissolution process with low to non-degraded silk fibroin material. This method is based on the findings of Chung et al. that the anionic detergent sodium dodecyl sulfate (SDS) improves the degree of degumming [21] and the hypothesis that reducing the cooking time by using microwave radiation reduces degradation of the silk during the degumming process.

Another objective of this work was the reduction of the required time and temperature for the complete dissolution of the degummed silk fibroin, with the aim of degrading the silk fibroin as little as possible. The method developed is originated from findings in the field of quality control of silk fibers. In 1935, Trotman and Bell described a method of detecting damage in silk by measuring the viscosity of a solution of silk in zinc chloride (ZnCl_2_) (67% *w*/*w*) after incubation for 6 h for 37 °C [22]. On the basis of these findings, it was shown later that the incubation time of silk in ZnCl_2_ could be reduced to 3 h by increasing the temperature to 45 °C [23].

However, so far, ZnCl_2_ has neither been used to isolate silk fibroin from degummed silk fibers nor has it been used to generate regenerated silk fibroin solution suitable for application as a biomaterial. Thus, a process will be presented, which is less time-consuming than the most commonly published methods.

## 2. Results

### 2.1. Degumming of Silk Fibers

**Figure 1:**
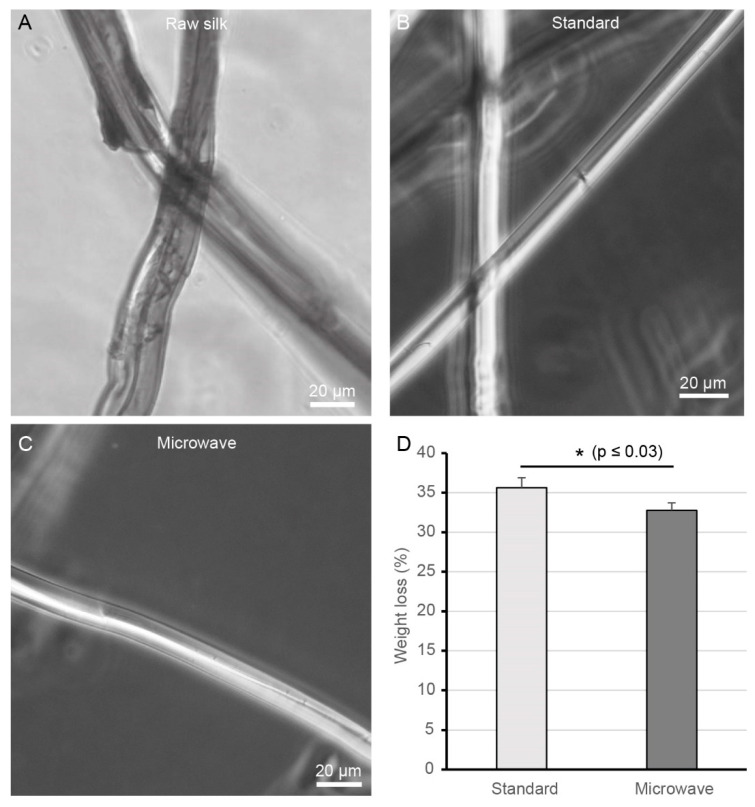
Light microscopic images of silk fibroin samples: (**A**) Raw silk fibers consist of two silk fibroin fibers covered and glued by a layer of sericin. (**B**) Silk fibroin fibers after standard degumming (30 min boiling in 0.02 M Na_2_CO_3_) and (**C**) microwave degumming. The scale bar corresponds to 20 µm. (**D**) Mean weight loss after degumming. The weight loss after degumming is expressed as the mean of four independent experiments ± standard error of the mean (SEM).

The untreated raw silk filament consists of a sericin layer that coats two fibroin filaments like an adhesive layer. This sericin layer appears as an uneven coating on the surface of the filaments, as shown in Figure 1A. During degumming of the silk, the sericin is completely removed from the fibroin filaments in the best case. In this study, a novel degumming method was compared with the most used method in the laboratory, boiling in Na_2_CO_3_ for 30 min (standard degumming), in terms of degumming efficiency and effects on silk fibroin structure. First, a qualitative evaluation of degumming efficiency was performed using microscopic images. The silk fibroin samples degummed by the standard method were perfectly smooth and thus completely freed from sericin. Furthermore, no remnants of the previous sericin layer could be observed (Figure 1B). Degumming with microwave treatment showed a comparably good picture in terms of degumming efficiency and also the silk fibroin fiber showed a smooth surface (Figure 1C). In addition, no obvious signs of damage to the fiber surfaces in the degummed silk fibroin samples from either method were visible under the microscope.

Another parameter of degumming efficiency is the determination of the weight loss of the degummed silk fibers. A comparison of the weight loss after treatment of silk samples with both degumming methods shows a higher weight loss of 35.6% for the standard degumming method. The microwave method presented, on the other hand, shows a weight loss of 32.8% (Figure 1D). Silk has a sericin content of 25–30% *w*/*w*. The observed higher mass loss beyond 30% is due to the washing step. During washing, the degummed silk fibers are held in a fine sieve under running water. In the process, small amounts can get caught in the sieve or even fall through it. However, this is not the reason for the observed lower mass loss by microwave degumming, as the washing step was performed identically in both methods.

**Figure 2:**
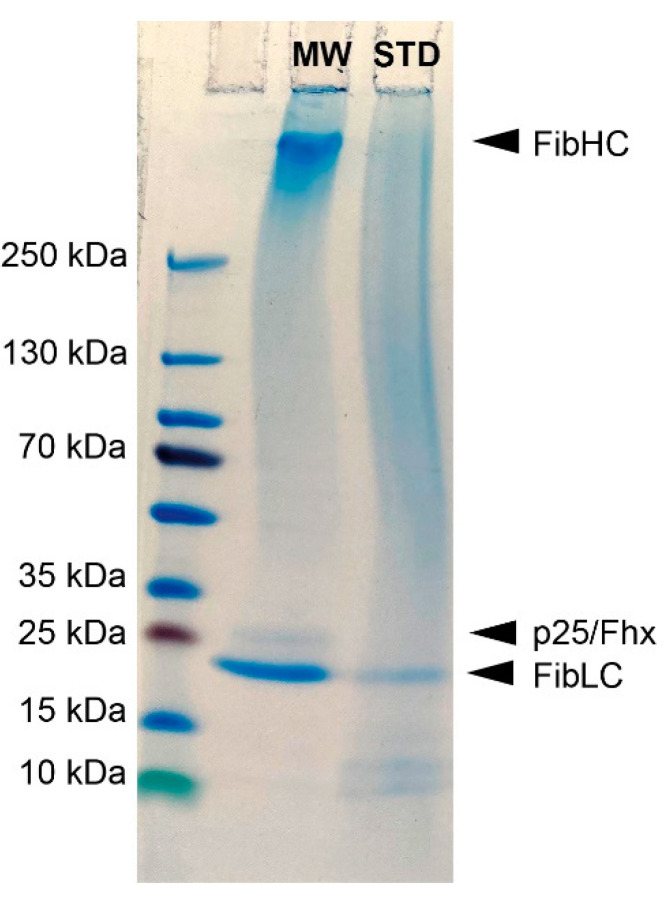
SDS-PAGE analysis of degummed silk fibers prepared by standard degumming (STD) or by microwave degumming (MW). Degummed fibers were dissolved in LiSCN and 40 μg of each silk fibroin solution was loaded on a precast gradient polyacrylamide gel (4–20%). PageRuler™ Plus Prestained Ladder (10 to 250 kDa) was used as a molecular mass marker. The molecular mass of the three subunits of silk fibroin (heavy chain fibroin, FibHC, 350 kDa; light chain fibroin, FibLC, 26 kDa; fibrohexamerin/p25, 30 kDa) are indicated by arrowheads.

To understand the effects of the two degumming methods on the molecular mass of silk fibroin and to investigate a possible destructive influence of the two methods, they were analyzed by SDS-polyacrylamide gel electrophoresis (SDS-PAGE) as shown in Figure 2. Samples of silk fibers degummed by either the standard method or the microwave method were dissolved in 70% lithium thiocyanate and separated electrophoretically in 4–20% Tris/glycine gels. When comparing the molecular masses of fibroin protein after each degumming, the microwave method (Figure 2, lane 2 MW) shows three clearly visible bands at 350 kDa, 30 kDa, and 25 kDa. In contrast, for the standard degumming method, only the band at 25 kDa and two others at 10 kDA and 12 kDa are visible. In the range from 130 kDa to 5 kDa, slight smearing is seen. Both indicate degradation of the silk fibroin and its subunits.

In summary, the degumming method presented in this study is as good as the standard method in terms of sericin removal efficiency. Furthermore, in contrast to the standard method, the microwave method is significantly more benign and does not lead to the degradation of fibroin.

### 2.2. Generation of Regenerated Silk Fibroin

**Figure 3:**
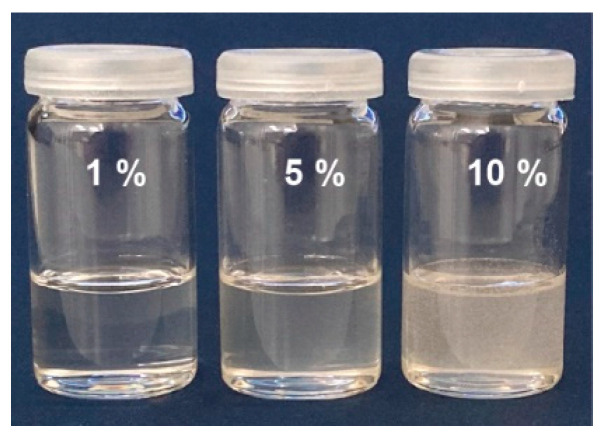
Photograph shows the appearance of different percentages of silk fibroin dissolved in ZnCl_2_ (56% *w*/*w*) after 1 h at 45 °C.

After developing a gentle degumming method that introduces less degradation to the silk fibroin structure than the standard degumming method, the next goal was to develop a process for producing a regenerated silk fibroin solution. Here, the main focus was on a short dissolution time combined with a low incubation temperature in contrast to the two most used methods using Ajisawa’s reagent [18] or LiBr [19]. Based on the publications of Trotman and Bell [22] and Tweedie [23], ZnCl_2_ was investigated for its suitability as a solvent. First, 0.5 g of degummed silk was incubated in 5 mL of ZnCl2 (56% *w*/*w*) at 45 °C for one hour with constant stirring. This resulted in a clear solution (Figure 3). The next step was to increase the amount of silk. Here it was found that it is possible to completely dissolve up to 10% degummed silk in a ZnCl_2_ solution (56% *w*/*w*) within one hour at 45 °C with constant stirring. It can be observed that the solution changes from translucent to a slightly cloudy and yellowish appearance with increasing silk fibroin concentration (Figure 3). In the case of the 10% (*w*/*v*) concentration of silk fibroin in ZnCl_2_, it was not investigated further whether this was a suspension or a complete dissolution. This will be the subject of future studies.

The most time-consuming step during the generation of regenerated silk fibroin in the most used methods is the desalting of the silk fibroin solution by dialysis. For this reason, the suitability of gel filtration as a desalting method was investigated in this study. For this purpose, 5 mL of a 1% silk solution was applied to desalting columns with an exclusion limit of 7 kDa. The silk/ZnCl_2_ solution migrates completely into the gel bed. While the ZnCl_2_ migrates into the pores of the column matrix, the molecules of the silk fibroin remain outside the gel matrix and therefore migrate through the column faster than the ZnCl_2_. Thus, when the silk is eluted with 7 mL of water, the silk fibroin is freed from the salt. Due to the high ZnCl_2_ concentration in the solution (56% *w*/*w*), the desalting step had to be repeated with a second gel filtration column. After the two desalting steps, the eluted silk fibroin solution no longer contains ZnCl_2_ as confirmed by the colorimetric assay kit for zinc. Subsequently, 1 mL of the eluted and desalted silk fibroin solution was dried and the resulting film was weighed. Then, based on the original amount of silk fibroin used and the dry weight of the silk fibroin film after desalting, the percentage recovery was calculated (Table 1). The results show an average recovery rate of 65 ± 2.6% of the amount of silk fibroin originally dissolved. In addition, the silk fibroin solution can be stored without gelation for at least 1 month at 4 °C (data not shown).

### 2.3. Silk Fibroin Film Morphology

**Figure 4:**
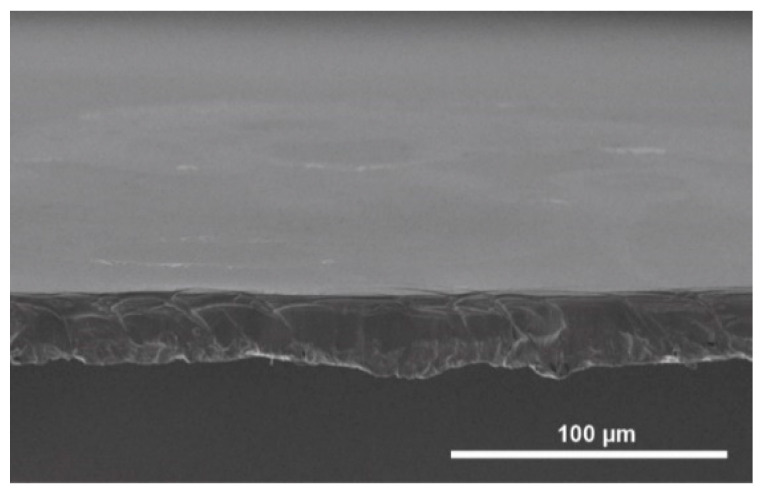
Scanning electron microscopy was applied to analyze the morphology of silk fibroin film cast from silk fibroin dissolved in ZnCl_2_ and desalted by gel filtration.

The morphology of films cast from regenerated silk fibroin, which was isolated by ZnCl_2_ dissolution and gel filtration was analyzed by scanning electron microscopy. These films show a very smooth surface and a homogeneous and uniform structure (Figure 4).

**Figure 5:**
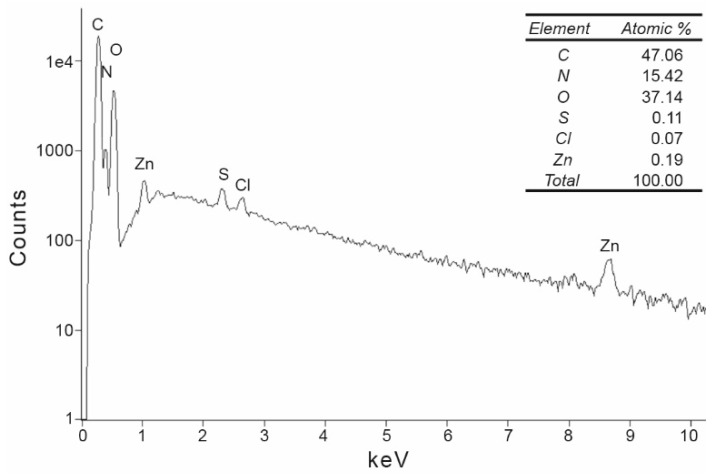
Energy-dispersive X-ray analysis (EDX) pattern of a silk film cast from silk fibroin dissolved in ZnCl_2_ and desalted by gel filtration. Data was recorded using an area analysis with a measuring area of 1 mm^2^. The inserted table presents the atomic percentage of the detected atoms.

To measure any residual ZnCl_2_ remaining on the silk films and to verify the data from the colorimetric test kit for water analysis for zinc, silk films made from regenerated silk fibroin were examined by energy-dispersive X-ray spectroscopy (EDX) analysis (Figure 5). The EDX spectra of these analyses show peaks for carbon, nitrogen, and oxygen, which are commonly associated with elements in proteins. However, there are also traces of sulfur, chlorine, and zinc. Thus, even the residual amount of zinc is only 0.19 atomic percent, zinc is still detectable on the film samples examined. Whether this very small residual amount of Zn has an influence on cytocompatibility will be investigated during the course of this study.

### 2.4. Attenuated Total Reflection Infrared Spectroscopy (ATR-FTIR)

**Figure 6:**
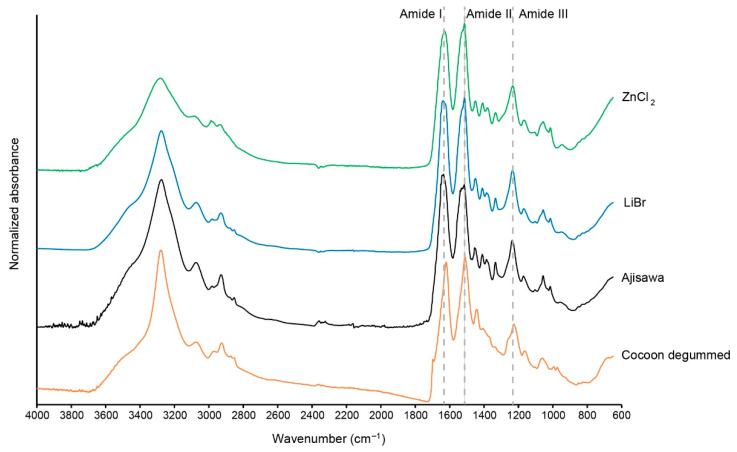
ATR-FTIR spectra of silk fibroin plotted as normalized absorbance versus wavenumber. Silk fibroin films cast from fibroin isolated using the three dissolution methods (Ajisawa, black; LiBr, blue; ZnCl_2_, green) were compared with degummed cocoon silk fibroin (yellow). Dashed lines show the bands for the three amides I-III.

To investigate the effects of the dissolution process in ZnCl_2_ on the silk fibroin protein, the infrared absorption spectra of silk fibroin membranes generated by different regeneration methods were analyzed. For comparison of the different spectra of silk films from the three different dissolution methods, degummed silk fibers were also examined and shown for reference purposes (Figure 6, yellow). All samples show strong absorption bands in the region of the characteristic fibroin features including the amide bands I-III, each representing mainly a mode of vibration C=O stretching, N-H deformation, and C-N stretching/N-H bending, respectively (Table 2) [14,24]. The three different silk membranes showed similar infrared absorption bands in amides I, II, and III in the range of 1633–1640 cm^−1^ (amide I), 1515 and 1516 cm^−1^ (amide II), and 1233–1237 cm^−1^ (amide III) regardless of the solution method (Figure 6, Ajisawa: black, LiBr: blue, ZnCl_2_: green, and Table 2). In this context, the infrared spectra and the bands specific to silk fibroin do not differ regardless of the dissolution method. Thus, dissolution of the silk fibroin in ZnCl_2_ represents a method equivalent to the two most commonly used dissolution methods. Only the degummed silk fibroin fibers (yellow) exhibit another specific vibration in the range of 1698 cm^−1^.

**Figure 7:**
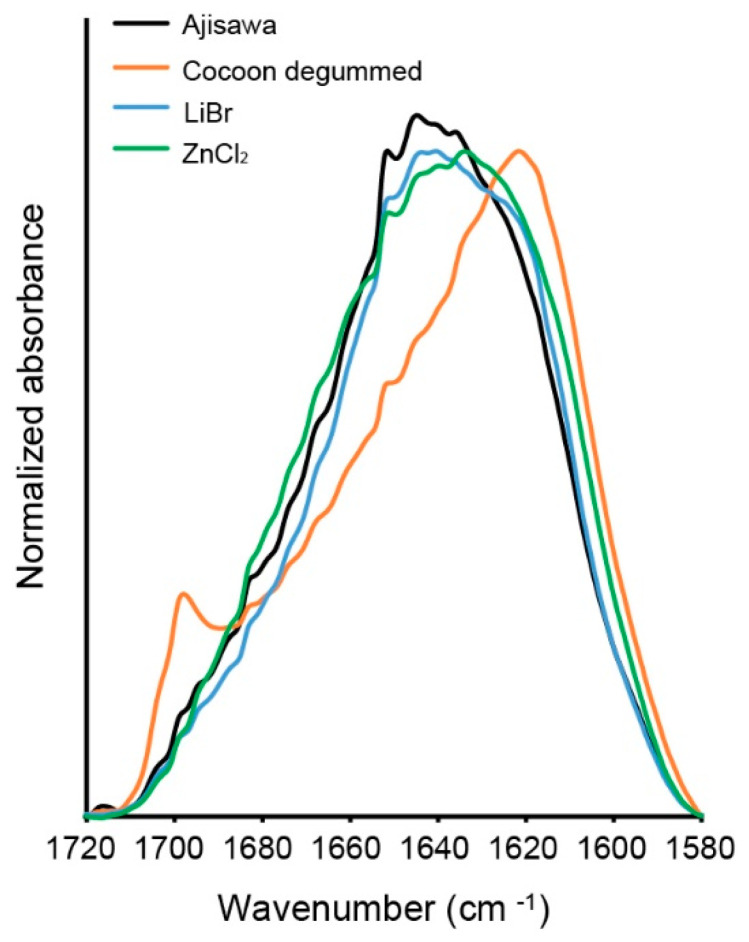
Baseline corrected ATR absorbance spectrum of the amide I band.

The amide I band can be used as a marker for the secondary structure of proteins, therefore only the amide I region from 1720 cm^−1^ to 1580 cm^−1^ is compared in Figure 7. Although the samples show similar characteristic profiles, some differences can also be seen. Silk fibroin from degummed cocoons shows amide I bands at 1700 cm^−1^ and at 1620 cm^−1^. These bands are due to dipole coupling and band splitting. This is observed when antiparallel beta-sheets are present. The bands that characterize the amide I band profile of silk fibroin polymorphs have been described elsewhere [15]. All three regenerated silk fibroin materials show enhanced random coil structures around 1640 cm^−1^ to 1625 cm^−1^ (Figure 7).

### 2.5. SDS-Polyacrylamide Gel Electrophoresis (SDS-PAGE)

**Figure 8:**
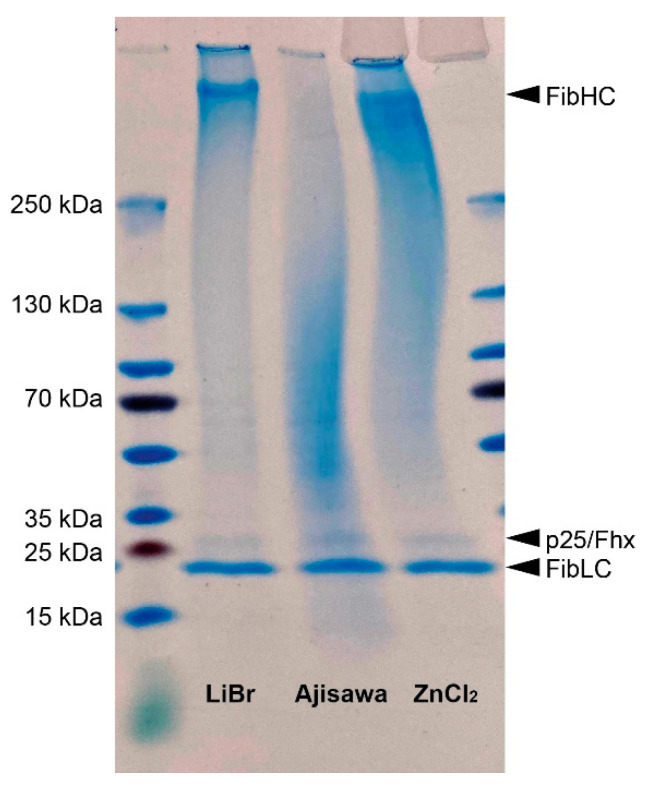
Comparison of molecular mass distribution of silk fibroin depending on isolation method by SDS-PAGE analysis. Films of all fibroin dissolved in LiBr, in Ajisawa’s reagent, or in ZnCl_2_ were dissolved in LiSCN and 40 μg of each solution was loaded on a precast gradient polyacrylamide gel (4–20%). PageRuler™ Plus Prestained Ladder (10 to 250 kDa) was used as a molecular mass marker. Molecular mass of the three subunits of silk fibroin (heavy chain fibroin, FibHC, 350 kDa; light chain fibroin, FibLC, 26 kDa; fibrohexamerin/p25, 30 kDa) are displayed by arrowheads.

The influence of the solvent ZnCl_2_ on the molecular structure of the silk protein or its subunits was analyzed by SDS-PAGE. For this purpose, films of silk fibroin dissolved using LiBr, Aijawa’s reagent or ZnCl_2_ were re-dissolved and separated by gel electrophoresis (Figure 8). Silk fibroin solubilized with LiBr shows three distinct bands that can be assigned to the three subunits of silk fibroin heavy chain (FibHC, 350 kDa), fibroin light chain (FibLC, 26 kDa), and fibrohexamerin/p25 (p25/Fhx, 30 kDa) (Figure 8, lane 1). In contrast, the band for the FibHC molecule is missing in the lane with silk fibroin isolated with Ajisawa reagent. Furthermore, a smear is visible between 130 kDa and 35 kDa. This indicates degradation of the FibHC molecule (Figure 8, lane 2). For silk solubilized with ZnCl_2_, a similar picture as for silk solubilized with LiBr is seen. Again, there are three distinct bands corresponding to the three subunits of fibroin (Figure 8, lane 3). These results show that ZnCl_2_ is a similar suitable solvent for silk fibroin and does not degrade the subunits of the silk fibroin molecule like with Ajisawa’s reagent. However, it must be noted that prolonged storage of the dissolved silk fibroin in the ZnCl_2_ solution without desalting leads to degradation of the fibroin molecule, which is additionally accompanied by lower viscosity (data not shown).

### 2.6. Direct Cytocompatibility

**Figure 9:**
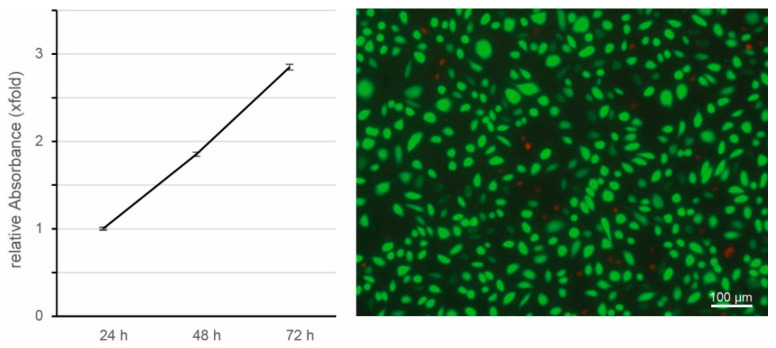
Cytocompatibility of silk fibroin films: **Left**: XTT assay for cell viability and proliferation of L929 cells cultivated on films from silk fibroin for 24 h, 48 h, and 72 h, respectively. The mean cell metabolic activity and standard error of the mean are reported for three independent silk fibroin samples dissolved with ZnCl_2_ in triplicates. **Right**: Representative fluorescence microscopy image of live-dead staining of L929 cells after 72 h cultivated on films from silk fibroin dissolved in ZnCl_2_.

The viability and proliferation of cells on films at different days are an indicator of cellular compatibility and suitability for use as a medical device or for tissue engineering applications. As a preliminary approach to evaluate the cytocompatibility of silk fibroin films, the XTT assay was performed. The increase in cell number from 24 h to 72 h after colonization of the films is linear and shows proliferation of L929 cells on the silk fibroin films (Figure 9 left). In addition, a live-death staining was performed after 72 h of cultivation. Evaluation of this staining shows that only very few cells are dead (red fluorescence) whereas most cells are alive (green fluorescence). Taken together, both results show that silk fibroin films do not induce cytotoxic effects on L929 cells cultured directly on the films.

## 3. Discussion

Since the most widely used degumming method in the literature is boiling silk fiber material in alkaline Na_2_CO_3_ solution for 30 min, this variant was used as the standard method in the present study. The standard degumming method has been shown to damage silk fibroin, as demonstrated by [10,11,25,26]. A moderate degumming method that does not damage silk fibroin is represented by the urea buffer developed by Yamada et al. However, this contains the toxic reagent β-mercaptoethanol as an essential component [3].

Therefore, in the present study, the standard method was modified to minimize the damaging effect. One alternative to conventional solution heating by boiling is microwave heating. By using microwave energy, faster, more uniform, and more efficient heating is possible. Microwave degumming in combination with Na_2_CO_3_ has already been used by Haggag et al. and Mahmoodi et al., respectively. However, with the treatment times applied there, only mass losses after degumming of 26% [27] or 23% [28] could be achieved. Furthermore, both sources show no data regarding a possible degradation of the silk fibroin. Therefore, the degumming time was reduced to four short one-minute repetitive microwave treatments. The anionic detergent SDS was added to the degumming solution in addition to the alkaline Na_2_CO_3_. As the first indication of a milder degumming, the determination of the mass loss after degumming showed a lower mass loss of 32.8% compared to a mass loss of 35.6% with the standard method (Figure 1). Since sericin accounts for 25–30% of the silk [29], it can be assumed that the sericin was completely removed. The increased mass loss in the standard method already indicates a possible loss or degradation of silk fibroin. The damage to silk fibroin by the standard degumming method is confirmed by the result of the SDS page analysis. This clearly shows that the fibroin heavy chain subunit was completely degraded by boiling the silk in an alkaline Na_2_CO_3_ solution for 30 min (Figure 2). Thus, the presented degumming method with repeated short-time microwave-induced boiling phases of 1 min in alkaline Na_2_CO_3_ solution with the addition of SDS is clearly gentler than the standard method and allows a degradation-free removal of sericin.

Once the silk fibroin was in an undamaged form after degumming, the next goal was to optimize the dissolution process in terms of the necessary temperature and the incubation time in the solvent. Here, the temperature was kept as low as possible and, at the same time, the incubation time was reduced so that the silk fibroin was damaged as little as possible. In addition, the amount of salt required in the solvent should be minimized to facilitate subsequent removal. The basis for the dissolution experiments were the publications showing good dissolution behavior of silk in highly concentrated ZnCl_2_ solutions [22,23]. The dissolution procedure adopted here, using 56% (*w*/*w*) ZnCl_2_ and incubating the degummed silk for 1 h at 45 °C, was able to dissolve degummed silk fibroin to a maximum of 10% (*w*/*v*) (Figure 3). The fact that ZnCl_2_ is a similarly good solvent for degummed silk fibroin as concentrated solutions of lithium salts e.g., LiSCN and LiBr is in agreement with the statement of Sashina et al. that the solubility of aqueous salt systems depends on the salt concentration and increases in the following order (for cations: Ca^2+^ < Sr^2+^ < Ba^2+^ < Li^+^ < Zn^2+^; for anions: Sulfate < Citrate < Tartrate < Acetate < Chloride < Nitrite < Bromide < Iodide < Thiocyanate < Dichloroacetate) [30].

For further analysis of the influence of ZnCl_2_ solvent on the structure of silk fibroin, the ZnCl_2_ silk fibroin solution had to be desalted first. The goal was to replace the time-consuming dialysis step with a less time-consuming but equally efficient desalting step. For this reason, the suitability of gel filtration (or size exclusion chromatography) for desalting the ZnCl_2_ silk fibroin solution was investigated. Gel filtration is suitable for many of the same purposes as dialysis since both methods exclude molecules based on their size. Compared to dialysis, gel filtration has the advantage of speed (several minutes versus hours for dialysis). In addition, gel filtration is compatible with organic and other solvents that dissolve or otherwise compromise the integrity of dialysis membranes. For example, ZnCl_2_ solutions in the concentration range of 60–70% (*w*/*w*) can dissolve cellulose [31] and thus dialysis membranes made of this material. Gel filtration has already been used to analyze the distribution of the molecular masses of silk fibroin after dissolution with LiBr and served only as an alternative method for SDS-PAGE and not for desalting. Rather, LiBr was previously removed by dialysis and only the aqueous silk fibroin solution was examined [32,33]. In contrast, silk fibroin dissolved in Ajisawa’s reagent (Ca^2+^/C_2_H_5_OH/H_2_O) has already been successfully desalted using gel filtration and the Ca^2+^ could be completely removed by this method [34,35,36].

In the present study, single-use columns were used for desalting, where the solution to be desalted flows through the column by gravity alone. It was found that a single pass was not sufficient to completely desalt the silk fibroin. For this reason, the eluate from the first pass was desalted a second time via a column. After the second run, the silk no longer contained ZnCl_2_ and was in an aqueous solution. The recovery yield of the silk fibroin was 65%. This is lower than the value obtained by Yeo et al. for the desalting of silk fibroin dissolved in Ajisawa’s reagent. Here, the recovery of silk fibroin protein during the desalting process was 85–90%, however, using a high-performance liquid chromatography (HPLC) system [35]. By using such a system, it is most likely that the recovery efficiency from the ZnCl_2_ solution can be further increased. In addition, the simultaneous use of a UV-Vis sensor and a conductivity detector can more accurately determine when the protein fraction is eluted from the column and when ZnCl_2_ elution begins. Together, this will further increase the yield of the silk fibroin protein fraction. However, the desalting method presented using single-use columns and gravity removes the ZnCl_2_ from the silk fibroin solution. Furthermore, the time required for desalting is only 3 h compared to dialysis, which takes at least 48 h.

The next step was to investigate the suitability of the regenerated silk fibroin isolated with ZnCl_2_ to produce silk films. The experiments showed that the isolated silk fibroin in an aqueous solution can be cast into films (Figure 4) and contains only very small amounts of ZnCl_2_ (0.19 atomic percent, Figure 5). However, these very small residual quantities of ZnCl_2_ have no influence on cell compatibility, as shown by the results of the cytotoxicity tests (Figure 9). The cells adhere and proliferate on the silk fibroin isolated with ZnCl_2_. The examination of the cells in direct contact for 72 h shows no evidence of a cytotoxic effect of the films.

Hereafter, the regenerated silk fibroin solution from the three solvent variants was investigated by ATR-FTIR to clarify a possible influence of ZnCL_2_ on the structure of the silk fibroin molecule. A comparison of the spectra of the ATR-FTIR analysis shows that the silk fibroin dissolved with ZnCl_2_ does not differ from the fibroin dissolved with standard solvents (LiBr, Ajisawa). The bands for amides I, II, and III are in the same range for all solution variants studied. Therefore, based on the position of the amide bands, the three spectral patterns studied can be attributed to a silk fibroin material with β-sheet molecular conformation. The band detected in the spectrum of the studied silk fibroin fibers at 1698 cm^−1^ is due to the antiparallel arrangement of the fibrin chains in the fold-sheet domains of the silk filament [14].

Finally, the results of the SDS-PAGE demonstrated that the ZnCl_2_ method presented has no effect on the structural integrity of the fibroin molecule (Figure 8) and no degradation occurs. The fibroin protein consists of one heavy chain molecule (350 kDa) [37] and one light chain molecule (26 kDa) [38]. Both molecules are linked by a disulfide bond between two cysteine amino acids to form a heterodimer. Five other heterodimers of heavy and light chain molecules are linked by a protein called fibrohexamerin/p25 (30 kDa, fhx/p25) [39] to form a so-called elemental unit in a molar ratio of 6:6:1 [40,41]. As with fibroin isolated by the LiBr method, all three subunits of the molecule isolated by the ZnCl_2_ method are intact and readily identifiable. In contrast, the Ajisawa method degrades the silk fibroin molecules [42].

**Figure 10:**
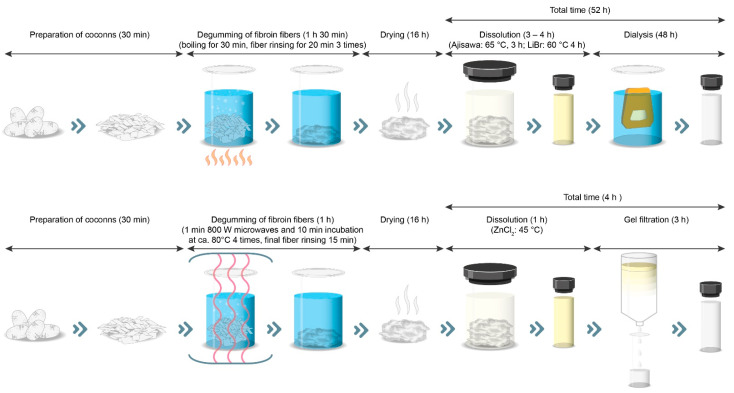
Comparison of the standard process for the production of regenerated silk fibroin (**above**) with the presented ZnCl_2_-based faster process (**below)**.

Thus, the new process presented here for generating regenerated silk fibroin solution is an alternative to standard methods that is also significantly faster to perform (Figure 10). The reason for this is the use of gel filtration instead of dialysis.

However, the enormous time savings of 48 h are contrasted by higher costs for the purchase of the gel filtration columns. Despite a lower price for ZnCl_2_ compared to LiBr (factor 3), the cost of isolating 15 mL of silk solution with the presented new method with ZnCL_2_ and gel filtration is about 1.9 times higher than the cost of the two standard methods. However, this consideration does not include the personnel costs. Due to the enormous time savings, it can be assumed that the personnel costs would be lower, which would put the higher material costs into perspective. The use of HPLC or FPLC equipment could also make desalination more automated and thus enable the generation of regenerated silk solutions on an industrial scale. In addition, the lower water consumption in desalination by gel filtration is an important aspect. For example, in ZnCl_2_ desalination by gel filtration, consumption of 200 mL compares to consumption of 6000 mL of water in dialysis. In addition, today there are effective methods for removing zinc from aqueous solutions such as ion exchange, chemical precipitation, chemical oxidation/reduction, electrodialysis, reverse osmosis, and ultrafiltration, which can thus further reduce water consumption.

## 4. Materials and Methods

### 4.1. Materials

Cocoons of the silkworm *Bombyx mori* were provided by the Council for Agricultural Research and Economics, Research Centre for Agriculture and Environment, Sericulture Laboratory, Padova, Italy. Unless otherwise stated, all chemicals utilized in this study were purchased from Carl Roth (Karlsruhe, Germany).

### 4.2. Silk Degumming

For degumming, *Bombyx mori* cocoons were cut into small pieces and 2 g cocoon pieces were heated for 1 min in 125 mL of an aqueous solution of 0.02 M Na_2_CO_3_ (Grüssing, Filsum, Germany) and 0.25% sodium dodecyl sulfate (SDS) in a 500 mL Duran^®^ bottle in a microwave (Clatronic MW 749, Clatronic International GmbH, Kempen, Germany) at 800 W. After microwave treatment the solution containing the silk was left for 10 min at room temperature. The temperature of the solution during this time was at 80 °C. Afterwards, the silk fibers were rinsed for 1 min with distilled water. Excess water was removed by squeezing the silk fibers. Then, after 3 additional cycles (microwave heating in fresh degumming solution, 10 min rest, washing and squeezing) the degummed silk fibers were dried overnight or directly used for dissolution.

In the standard degumming method, 2 g cocoon pieces were boiled under constant stirring in 1 L of 0.02 M Na_2_CO_3_ (Grüssing, Filsum, Germany) for 30 min and rinsed 3 times for 20 min [19].

Degumming efficiency was measured by determining the percentage weight loss of samples after degumming. It was expressed as a percentage of the initial weight. All the degummed samples were evaluated with an optical microscope (AxioVert.A1, Carl Zeiss, Jena, Germany) to assess the extent of degumming and fiber damage.

### 4.3. Dissolution of Silk Fibroin Fibers and Desalting

All dissolution experiments were carried out in a preheated oven with constant stirring. For this purpose, the solvent was always poured onto the silk previously placed in the vessel so that the silk was completely submerged in the solvent.

#### 4.3.1. Ajisawa’s Reagent (CaCl_2_/C_2_H_5_OH/H_2_O)

1 g of degummed silk fibers were incubated in 10 mL (10%, *w*/*v*) of CaCl_2_/EtOH/H_2_O (molar ration 1/2/8) (*v*/*w*) at 65 °C for 3 h [18]. For desalting, the silk fibroin solution was dialyzed, using a Membra-Cel™ regenerated cellulose membrane with a Molecular Weight Cut-Off of 14,000 Dalton. Stepwise dialysis against urea solution was performed to avoid fibroin aggregation [43]. The dialysis tubes were placed in 100 parts of 4 M urea solution for 3 h with slow stirring, followed by changing the solution to 100 parts of 2 M and 1 M urea for 3 h, respectively, followed by a final dialysis step against water overnight and until the conductivity was below 10 µS.

#### 4.3.2. Lithium Bromide (LiBr)

1 g of degummed silk fibers were incubated in 10 mL (10%, *w*/*v*) of 9.3 M LiBr at 60 °C for 4 h. The silk fibroin solution was dialyzed, using a Membra-Cel™ regenerated cellulose membrane with a Molecular Weight Cut-Off of 14,000 Dalton and direct dialysis against water for 48 h or until the conductivity was below 10 µS.

#### 4.3.3. Zinc Chloride (ZnCl_2_)

0.1 g of degummed silk fibers were incubated in 10 mL of 56% (*w*/*w*) aqueous ZnCl_2_ solution at 45 °C for 1 h, yielding a 1% (*w*/*v*) silk fibroin solution. For desalting gel filtration was applied. Gel filtration columns were used to separate ZnCl_2_ from silk fibroin according to the manufacturer’s instructions. In brief, 5 mL of a Silk/ZnCl_2_ solution was transferred to an equilibrated CentriPure P50 Column (Serva, Heidelberg, Germany) and allowed to enter the gel bed completely. The gel matrix in CentriPure P50 is Zetadex-25, a size-exclusion gel with an effective pore size of about 5 kDa. Then, 7 mL of water was used to elute the sample into a collection tube. After one round the eluted silk solution still contained too much ZnCl_2_, so the procedure was repeated with a second column. The absence of zinc within the eluted silk fibroin solution was determined semiquantitatively using the MQant^®^ Zinc Test (Merck, Darmstadt, Germany).

For all three kinds of silk fibroin concentration was calculated by weighting the residual solid of a known volume of silk fibroin solution after drying and by division of the determined weight by the volume before drying.

### 4.4. Preparation of Silk Fibroin Films

Silk fibroin films were cast on the surfaces of polystyrene weigh boats directly from the solution and allowed to dry. Exclusively for cell culture experiments, silk fibroin films were treated with 90% methanol for 30 min to make the films insoluble in water followed by three washing steps with phosphate-buffered saline (PBS) for 15 min each. For all other experiments with silk films, untreated films were used.

### 4.5. SDS-Polyacrylamide Gele Electrophoresis (SDS-PAGE)

The molecular mass distribution of silk fibroin independence on the degumming and dissolution conditions used was studied with SDS-PAGE. Silk fibroin films were dissolved in LiSCN (70%) overnight at room temperature at a concentration of 10 µg µL^−1^. For each sample, 30 µg were loaded on a precast vertical 4–20% Tris/Glycine gel (SERVAGel™ TG Prime™) (SERVA, Heidelberg, Germany) under reducing conditions in Tris/Glycine SDS Buffer (Fisher Scientific, Schwerte, Germany). SDS-PAGE was run at 250 mA with a molecular mass ladder (PageRuler™ Plus Prestained Protein Ladder, 10 to 250 kDa) (Fisher Scientific, Schwerte, Germany) and then stained with a colloidal Coomassie staining solution (Quick Coomassie^®^ Stain) (SERVA, Heidelberg, Germany).

### 4.6. Attenuated Total Reflection Infrared Spectroscopy (ATR-FTIR)

ATR-FTIR spectra of silk fibroin films cast from the different applied solvents were recorded using a Nicolet 6700 spectrometer (Thermo Fisher Scientific, Waltham, MA, USA). The spectrometer was equipped with a single-bounce diamond ATR accessory (Smart iTR, Thermo Fisher Scientific, Waltham, MA, USA) with a refractive index of 2.4 and an active sample area diameter of 1.5 mm. Spectra of each of the samples were acquired by pressing the films on the ATR crystal. All samples were measured in reflection mode. For all measurements the following parameters were used: (i) resolution 4 cm^−1^, (ii) spectral range 650–4000 cm^−1^, (iii) apodization window Norton and Beer (strong), and (iv) the number of scans 64.

### 4.7. Scanning Electron Microscopy (SEM) and Energy Dispersive X-ray (EDX) Spectroscopy

Silk fibroin film morphology was examined by SEM. Samples of dried films were sputter-coated with gold using a Cressington 108auto sputter coater (TESCAN, Dortmund, Germany) and imaged with a Quanta 250 FEG ESEM (Thermo Fisher Scientific, Waltham, MA, USA).

EDX spectroscopy was used to detect residual zinc ions in the silk fibroin films. Graphite coating of SEM samples for the deposition of a conducting layer was performed using a sputtering process. EDX spectra and elemental maps were obtained by acquiring area scans using a Zeiss DSM 950 scanning electron microscope (Carl Zeiss, Jena, Germany) with a Noran System 7 EDX with an SDD detector (Thermo Fisher Scientific, Waltham, MA, USA).

### 4.8. In Vitro Cytotoxicity

Possible cytotoxic effects of the silk fibroin isolated with ZnCl_2_ were analyzed by a direct cell contact assay using the mouse fibroblast cell line L929 (German Collection of Microorganisms and Cell Cultures, DSMZ, Braunschweig, Germany). L929 cells were cultured in Roswell Park Memorial Institute 1640 (RPMI 1640) medium (Lonza, Basel, Switzerland) containing 10% fetal bovine serum (Fisher Scientific, Schwerte, Germany), 2 mM Glutamax (Lonza, Basel, Switzerland) and 1.0% (*v*/*v*) penicillin/streptomycin (Lonza, Basel, Switzerland). For direct contact assay 200 µL of the silk fibroin solution was added into wells of a 96 well cell culture plate (Sarstedt, Nümbrecht, Germany), dried overnight and incubated in 200 µL of 90% methanol for 30 min. Then, silk fibroin coated wells were washed 3 times for 15 min each with Phosphate Buffered Saline (PBS) without calcium and magnesium (Lonza, Basel, Switzerland) and seeded with 2.5 × 10^4^ cell in 100 µL RMPI 1640 medium, without phenol red supplemented with 10% fetal bovine serum, 2 mM Glutamax and 1.0% (*v*/*v*) penicillin/streptomycin. Cells were cultured for 24, 48 and 72 h in a CO_2_-incubator.

The XTT Cell Proliferation Assay Kit (Serva, Heidelberg, Germany) was used to quantitatively evaluate cell metabolic activity according to the manufacturer’s protocol after 24, 48, and 72 h, respectively. The electron coupling and XTT labeling reagents were thawed and immediately combined in a 1 μL:50 μL ratio. Then 50 µL of this XTT solution was added to the cell culture wells of the 96 well plate and mixed gently. The absorbance signal of the samples was measured after 2 h of incubation at 37 °C with a Multiskan FC microplate photometer (Fisher Scientific, Schwerte, Germany) at a wavelength of 450 nm and for background absorbance at a wavelength of 650 nm. Normalized absorbance values were calculated by subtraction of background absorbance from signal absorbance. Samples were evaluated, the mean cell metabolic activity and standard error of the mean are reported for three independent silk fibroin samples dissolved with ZnCl_2_ in triplicates.

After 72 h cultivation of the L929 cell on silk fibroin dissolved by ZnCl_2_, live and dead cells were visualized after the staining of cells with fluorescein diacetate (FDA) and propidium iodide (PI) (Merck, Darmstadt, Germany). In living cells, FDA is hydrolyzed by intracellular esterases and thus, viable cells will appear green fluorescent when excited with light in the range of 450–490 nm. PI can only enter dead or dying cells. It binds to nucleic acids and causes them to fluoresce red after excitation by 530−585 nm. For live and dead staining cell culture medium was replaced by 100 µL of staining solution consisting of 0.8 µg FDA and 2 µg PI in serum-free RPMI 1640. Cells were incubated at room temperature for 5 min in the dark. Then, the staining solution was removed, cells were washed with PBS, and finally, medium without FCS was added to the samples. Cells were analyzed with a fluorescent microscope (AxioVert.A1, Carl Zeiss, Jena, Germany).

### 4.9. Statistical Evaluation

Statistical analysis was conducted using one-way analysis of variance (ANOVA) followed by Student’s *t*-test (independent, two-sided). Differences were considered significant for *p* < 0.05. All data are expressed as means ± standard error of the mean (SEM).

## 5. Conclusions

In this study, a new method for the preparation of regenerated silk fibroin solution is presented that is feasible in significantly less time than the most widely used methods in the literature (4 h versus 52 h). The use of a gentle degumming method, the use of ZnCl_2_ as a solvent, and subsequent desalting by gel filtration allows the preparation of a cytocompatible silk fibroin solution without compromising the structural integrity of the fibroin molecule. Therefore, the presented method represents a step towards the isolation of native-like silk fibroin and could be used in the future for the production of regenerated silk fibroin on an industrial scale.

## Figures and Tables

**Table 1:** Recovery rate of silk fibroin after dissolution in ZnCl_2_ (56% *w*/*w*) and desalting using gel filtration.

Sample	Recovery Rate (%)	Residual ZnCl_2_ (µg/mL)
1	57.3	0
2	69.8	0
3	61.4	0
4	71.6	0
Mean ± SEM	65.0 ± 2.6	

**Table 2:** Comparison of main bands of ATR-FTIR spectra (cm^−1^) of silk fibroin films.

Sample	Random Coil Structure ^$^	β-Sheet ^$^	Ajisawa	LiBr	ZnCl_2_
Amide I (C=O stretching)	1640–1660	1625–1640	1636	1640	1633
Amide II (N-H deformation)	1540–1550	1520–1530 1515 ^§^	1516	1515	1515
Amide III (C-N stretching, N-H bending)	1257–1313	1219–1245	1237	1235	1233

References for Random coil and β-sheet: ^$^ [24], ^§^ [14].

## Data Availability

Not applicable.
